# The relationship between the use of mental health act and elderly suicide rates in England and Walls

**DOI:** 10.5249/jivr.v1i1.58

**Published:** 2009-07

**Authors:** Ajit Shah, Laura Buckley

**Affiliations:** ^*a*^University of Central Lancashire, Preston, United Kingdom and Consultant Psychiatrist, West London Mental Health NHS Trust, London, United Kingdom; ^*b*^Student Intern, International School for Communities, Rights and Inclusion, University of Central Lancashire, Preston, United Kingdom

## Abstract

**Background:**

The relationship between suicide and involuntary admissions has been examined in younger and mixed age groups. These studies provide mixed results with some demonstrating no relationship and others reporting increased rates of suicides in involuntarily admitted patients. This relationship has not been examined in the elderly.

**Methods:**

An ecological study, over the 19-year period, to examine the relationship between rates of involuntary admissions and elderly suicide rates in England and Wales was undertaken using nationally collected data. Data on suicide rates for both sexes in the age-bands 65-74 years and 75+ years were ascertained from the World Health Organization (WHO) website. Data on the number of detentions under the Mental Health Act were ascertained from the Office of National Statistics website. Data on the population size for the elderly age-bands were ascertained from the WHO website. Spearman's correlation coefficient was used to examine the relationship between suicide rates and rates of detention under the Mental Health Act.

**Results:**

There were negative correlations between rates of involuntary admissions and suicide rates in both sexes in the age-bands 65-74 and 75+ years.

**Conclusions:**

A causal relationship and the direction of causality cannot be assumed because this was an ecological study. There is a need for sufficiently powered study to compare the number of suicides occurring in involuntarily and voluntarily admitted patients using a case-control or cohort design and survival analysis. If an inverse association can be demonstrated between suicide and involuntary admissions then it has important implications for the development of mental health legislation as an adjunct to national suicide prevention strategies.

## Introduction

 Involuntary admission into hospital under relevant mental health legislation is generally used to protect patients or others from harm. In this context, individuals at potential risk of suicide may be involuntarily admitted.^1^ Moreover, there is an increased risk of inpatient suicide within the first seven days of involuntary inpatient admission.^2^

 A cross-national and ecological study of 100 countries reported that general population suicide rates increased after the introduction of mental health legislation.^3^ However, in another cross-national and ecological study, there was no significant association between presence of mental health legislation and elderly suicide rates.^4^ The mere presence of mental health legislation may not necessarily lead to consistent implementation of this legislation, and this may explain an absence of association between decline in suicide rates and implementation of mental health legislation in both these studies.^3,4^ It may be that the presence of mental health legislation is a proxy for higher needs, or that countries adopt these policies when the suicide rates are already increasing.

A Canadian study comparing the number of suicides between those with involuntary and voluntary psychiatric admissions at nine year follow-up found no difference between the two groups.^5^ A study of suicides within one year of contact with mental health services in England and Wales reported an increase in the number of suicides in those aged under 25 years, compared to older age groups, admitted involuntarily.^6^ However, this association was not observed in other English studies of psychiatric inpatients discharged and followed-up for one year,^7^ and two to twelve years.^8^ Another English study reported that the number of suicides during the admission were significantly higher in those involuntarily than voluntarily admitted,^9^ but this may reflect severity of illness requiring admission.^9^ Comparison between these different studies are difficult because of the different methodologies used.

With the exception of one study,^4^ all other studies have focused on either younger age groups or mixed age groups. Moreover, studies examining the number of suicides in involuntary and voluntary patients may miss an association because the sample size in most studies has been small and suicide is a comparatively rare event. The elderly are the group at the highest risk of suicide^10^. Moreover, over 90% of elderly suicide victims have symptoms of depression^10^. Furthermore, the relationship between elderly suicides and rates of detention under Mental Health Legislation has not been previously studied. Thus, an ecological study, over a 19-year period, to examine the relationship between rates of involuntary admissions and elderly suicide rates in England and Wales was undertaken. Given the mixed findings of an association in the literature, a null hypothesis that there will be no association between rates of involuntary admissions and elderly suicide rates was proposed.

## Methods

Data on suicide rates for males and females in the age-bands 65-74 years and 75+ years for England and Wales was ascertained from the World Health Organization (WHO) website (http://www.who.int/whosis/database/mort/table1.cfm) for each of the 19 years between 1988 and 2006.

Data on the number of detentions under the Mental Health Act 1983 (MHA1983) for all detentions, those under Sections 4, 2 and 3 of the MHA1983 and those under Part III of the MHA1983, were ascertained from the Office of National Statistics website (http://www.dh.gov.uk/en/Publicationsandstatis tics/Statistics/StatisticalWorkAreas/Statisticalhealthcare/DH_4086494) for each of the 19 years between 1988 and 2006. The Mental Health Act in England and Wales recommends that patients should generally be admitted under Section 2 (for assessment lasting 28 days) or Section 3 (for treatment lasting 6 months) of the Act, which requires two doctors and an approved mental health professional (e.g. a social worker) to complete. Section 4 can only be justified in emergencies when only one doctor is available, it lasts for only 72 hours, and either a Section 2 or Section 3 must be completed during this 72 hour period.

Data on the size of the general population in England and Wales were also ascertained from the WHO website (http://www.who.int/whosis/database/mort/table1.cfm) for each of the 19 years between 1988 and 2006.

The rates of detention under the MHA1983 for all detentions, detentions under Sections 4, 2 and 3 of the MHA1983, and detentions under Part III of the MHA1983 were calculated by dividing the number of detentions in each category by the size of the general population for each of the 19 years.

The relationship between suicide rates in both the elderly age-bands in both sexes and the rates of detention under the MHA1983 for each category of detention across the 19 years was examined with Spearman’s correlation coefficient (rho). This non-parametric test was used because the data on suicide rates are not normally distributed.

## Results

Full data sets were available for the 19-year period 1988 to 2006. Table 1 illustrates the median and range of suicides rates in both sexes in both the elderly age-bands for the 19-year period. Table 2 illustrates the relationship between suicide rates in both the elderly age-bands in both sexes and the rates of detention under the MHA1983 for each category of detention.

**Table:** Table 1: **Median and range of suicide rates(per 100,000 of the population in the relevant age and sex band)**

Group	Median	Range
Males aged 65-74 years	9.8	8.4-16.2
Males aged 75+ years	15.2	10.6-24.3
Females aged 65-74 years	4	2.7-8
Females aged 75+ years	5	3.1-8.4

Overall, suicide rates for both age-band and gender groups were negatively correlated with rates of detentions (P<0.0001). Further, when analyzed separately by sections, suicide rates in all four studied groups were found to have significant negative correlations with rates of detention under the Mental Health Act (aside from those detained under Section 4 and Part III of the MHA1983, which were not significant).

**Table:** Table 2: **The relationship between elderly suicide rates and rates of detention under the Mental Health Act 1983**

Rates of Detention	Suicide Rates			
	Males 65-74 Yrs	Males 75+ Yrs	Females 65-75 Yrs	Females 75+ Yrs
All Detentions	Rho=-0.79	Rho=-0.85	Rho=-0.77	Rho=-0.79
	P<0.0001	P<0.0001	P<0.0001	P<0.0001
Section 4	NS	NS	NS	NS
Section 2	Rho=-0.85	Rho=-0.91	Rho=-0.82	Rho=-0.82
	P<0.0001	P<0.0001	P<0.0001	P<0.0001
Section 3	Rho=-0.67	Rho=-0.68	Rho=-0.65	Rho=-0.72
	P=0.002	P=0.002	P=0.003	P<0.0001
Detention under Part III of MHA1983	NS	NS	NS	NS
NS= Not Significant								

## Discussion

There was no association between elderly suicide rates and involuntary admission under Section 4 of the Mental Health Act. The Mental Health Act in England and Wales recommends that patients should generally be admitted under Section 2 or Section 3 of the Act, which requires two doctors and an approved mental health professional (e.g. a social worker) to complete, and where possible to avoid involuntary admission under Section 4. Section 4 can only be justified in emergencies when only one doctor is available, it lasts for only 72 hours, and either a Section 2 or Section 3 must be completed during this 72 hour period. Consequently Section 4 is infrequently used, and this limited use may explain an absence of association with suicide rates.

There was no association between elderly suicide rates and involuntary admission under Part III of the Mental Health Act. This part of the Act refers to involuntary admissions into psychiatric hospitals through the criminal justice system. This part of the Act is rarely used with elderly patients,^11,12^ and this may explain the absence of an association.

There were negative correlations between suicide rates and rates of involuntary admission in both the elderly age-bands. This was true for both sexes, and overall rates of involuntary admission as well as admissions under Sections 2 and 3 of the Mental Health Act. These findings are consistent with those reported for younger and mixed age groups in some studies.^9^ However, they were not consistent with opposite findings in one study^3^ and the absence of an association in other studies,^5,7,8^ including a cross-national study of elderly suicides.^4^

The observed correlations in the current study may be due to methodological issues. Data were not specifically available for involuntary admission of elderly patients and, thus, data for the general population were utilized. The general population detention rates may not represent detention rates for older people. The proportion of involuntary admissions of older people under the Mental Health Act in England and Wales is higher than the proportion of older people in the general population.^1^ Thus, the current study may have under-estimated the rates of detention for older people. In England and Wales, the coroner can only return a verdict of suicide if suicide can be proved beyond reasonable doubt. Therefore, some genuine suicides may be misclassified as an open verdict when suicide cannot be proved beyond a reasonable doubt.^13,14^ This may have resulted in a lower actual elderly suicide rate being included in the analysis, but data on deaths due to open verdicts were not available from the WHO for the period before 2001 when the ICD-9 classification was used. It’s also possible that other factors may independently influence elderly suicide rates and rates of involuntary admission in opposite directions leading to spurious correlations (epiphenomena). It is also possible that the correlations are genuine and this is examined below.

If the results are genuine then there are two possibilities: first, reduced elderly suicide rates may lead to an increase in rates of involuntary admissions; and second, increased rates of involuntary admission may reduce elderly suicide rates. There is little theoretical, clinical or legal evidence to explain why lower elderly suicide rates would lead to an increase in rates of involuntary admissions. A better case can be made to explain why increased rates of involuntary admission may lead to lower elderly suicide rates. Rates of involuntary admissions may increase through several mechanisms. First, changes in mental health legislation may increase rates of involuntary admissions,^15^ but this had not occurred during the study period. Second, a range of national initiatives designed to reduce suicide rates may lead to an increase in rates of involuntary admissions, and these included: legislation requiring general practitioners to offer annual physical and mental examination to those aged over 75 years; the Defeat Depression Campaign organized by the Royal College of Psychiatrist; the National Confidential Enquiry into Suicides and Homicides; the governmental “Our Healthier Nation” suicide reduction targets; the National Service Frameworks for Mental Health and for Older People; and the National Suicide Prevention Strategy.^16,18^ Third, high profile investigation into suicides and high profile media reporting of suicides may also encourage clinicians to use mental health legislation for involuntary admissions more frequently. Fourth, increased rates of suicide may also encourage clinicians in increasing the use of mental health legislation for involuntary admissions; there is evidence of an association between mental health programs and policy and increased suicide rates in the general population.^3^ Increase in the rates of involuntary admissions is likely to afford close supervision and protection of suicidal patients during the admission and facilitate reduction in the number of suicides. Moreover, treatment in hospital given to these involuntarily admitted patients may also lead to improvement in their mental illness and further facilitate reduction in the number of suicides. This is particularly important in the elderly because up to 90% of elderly suicide victims have depressive symptoms,^10^ which is potentially treatable. However, the results should also be interpreted in the context of observations that the elderly suicide rates in England and Wales have been declining at least over the last 21 years.^18^

However, a causal relationship and the direction of causality cannot be assumed because this was an ecological study. There is, therefore, a need for sufficiently powered study to compare the numbers of suicide occurring in involuntarily and voluntarily admitted patients, over at least a ten year follow-up period, using a case-control or cohort design and survival analysis. If an inverse association can be demonstrated between suicide and involuntary admissions then this has important implications for the development of mental health legislation as an adjunct to national suicide prevention strategies.
